# Automatic Assessment of Emotion Dysregulation in American, French, and Tunisian Adults and New Developments in Deep Multimodal Fusion: Cross-sectional Study

**DOI:** 10.2196/34333

**Published:** 2022-01-24

**Authors:** Federico Parra, Yannick Benezeth, Fan Yang

**Affiliations:** 1 LE2I EA 7508 Université Bourgogne Franche-Comté Dijon France

**Keywords:** emotion dysregulation, deep multimodal fusion, small data, psychometrics

## Abstract

**Background:**

Emotion dysregulation is a key dimension of adult psychological functioning. There is an interest in developing a computer-based, multimodal, and automatic measure.

**Objective:**

We wanted to train a deep multimodal fusion model to estimate emotion dysregulation in adults based on their responses to the Multimodal Developmental Profile, a computer-based psychometric test, using only a small training sample and without transfer learning.

**Methods:**

Two hundred and forty-eight participants from 3 different countries took the Multimodal Developmental Profile test, which exposed them to 14 picture and music stimuli and asked them to express their feelings about them, while the software extracted the following features from the video and audio signals: facial expressions, linguistic and paralinguistic characteristics of speech, head movements, gaze direction, and heart rate variability derivatives. Participants also responded to the brief version of the Difficulties in Emotional Regulation Scale. We separated and averaged the feature signals that corresponded to the responses to each stimulus, building a structured data set. We transformed each person’s per-stimulus structured data into a *multimodal codex*, a grayscale image created by projecting each feature’s normalized intensity value onto a cartesian space, deriving each pixel’s position by applying the Uniform Manifold Approximation and Projection method. The codex sequence was then fed to 2 network types. First, 13 convolutional neural networks dealt with the spatial aspect of the problem, estimating emotion dysregulation by analyzing each of the codified responses. These convolutional estimations were then fed to a transformer network that decoded the temporal aspect of the problem, estimating emotional dysregulation based on the *succession* of responses. We introduce a Feature Map Average Pooling layer, which computes the mean of the convolved feature maps produced by our convolution layers, dramatically reducing the number of learnable weights and increasing regularization through an ensembling effect. We implemented 8-fold cross-validation to provide a good enough estimation of the generalization ability to unseen samples. Most of the experiments mentioned in this paper are easily replicable using the associated Google Colab system.

**Results:**

We found an average Pearson correlation (*r*) of 0.55 (with an average *P* value of *<*.001) between ground truth emotion dysregulation and our system’s estimation of emotion dysregulation. An average mean absolute error of 0.16 and a mean concordance correlation coefficient of 0.54 were also found.

**Conclusions:**

In psychometry, our results represent excellent evidence of convergence validity, suggesting that the Multimodal Developmental Profile could be used in conjunction with this methodology to provide a valid measure of emotion dysregulation in adults. Future studies should replicate our findings using a hold-out test sample. Our methodology could be implemented more generally to train deep neural networks where only small training samples are available.

## Introduction

Emotion regulation is currently conceptualized as involving the following 5 distinct abilities: (1) having awareness and an understanding of one’s emotions, (2) being able to accept them, (3) being able to control impulsive behaviors related to them, (4) having the capacity to behave according to our desired goals in the midst of negative emotions, and (5) having the capacity to implement emotion regulation strategies as required to meet individual goals and situational demands. The absence of these abilities indicates the presence of *emotion dysregulation* [1]. Psychopathology is characterized by intense or protracted maladaptive negative emotional experiences. Emotion dysregulation is a core vulnerability to the development of both internalizing and externalizing mental disorders [2]. For example, high emotion dysregulation is a key component of substance abuse [3], generalized anxiety disorder [4], complex posttraumatic stress disorder [5], and borderline personality disorder [6].

Emotion dysregulation is typically assessed through a self-report questionnaire, the Difficulties in Emotional Regulation Scale (DERS) [1], or one of its shorter forms (eg, Difficulties in Emotion Regulation Scale, brief version [DERS-16]) [7]. It can also be assessed physiologically by measuring heart rate variability (HRV) in a controlled experiment, with the advantage that this requires no insight from the participant and represents an objective measure. However, traditionally, this form of assessment represented serious costs of collection, and varying baselines among people posed a problem [8]. Since at least one study has shown that the DERS and the HRV-based assessment of emotion dysregulation are correlated [8], the DERS has become the de-facto “gold standard.”

Attempts to measure psychological dimensions “in the wild” (ie, a naturalistic approach) using machine learning and unimodal sensing approaches, such as measuring heart rate throughout the day with a smartwatch or measuring the patterns of social media interactions by a user, have not yet produced good enough results leading to major changes in the way the mental health industry practices psychometrics. It still relies almost entirely on self-assessment questionnaires or professional interviews [9]. In our view, this absence of disruption comes down to 2 issues. First, the problem of relying on a single modality. In the field of affective computing, multimodal fusion has shown promise by beating unimodal approaches in several benchmarks [10]. This is because multimodality provides cross-validation of hypotheses, where one sense modality can reaffirm or negate what was perceived by another, reducing error and increasing reliability. This is how we, humans, perceive. Second, measuring psychological dimensions “in the wild” might be a bad idea due to the unknown number of confounding factors surrounding daily life. In particular, many authors underline the need for considering the specific demands of the situation at hand, as well as the specific goals of the individual in that context, when evaluating emotion dysregulation [1].

To overcome these limitations, in 2017, we introduced the Biometric Attachment Test (BAT) in the Journal of Medical Internet Research [11]. It was and continues to be the first automated computer test to measure adult attachment in a multimodal fashion, including physiology measures (HRV) as well as behavioral ones. The BAT uses picture and music stimuli to evoke situations and feelings related to adult attachment, such as loss, fear, parent-children relationships, or romantic relationships. It sits well within the psychometric tradition of projective tests, such as the Thematic Apperception Test [12]. In 2019, we presented a machine learning methodology to automatically score the BAT using a small training data set, and we validated the use of a remote photoplethysmography (RPPG) algorithm to measure HRV in a contactless fashion as part of the BAT software [13]. We have now renamed our test to the Multimodal Developmental Profile (MDP), because we hypothesize its stimuli and design can work for measuring not only adult attachment, but also several other dimensions of psychological functioning that are developmental in nature and crucial to the forming of psychopathology [14]. In particular, we hypothesize that the MDP can measure emotion dysregulation in adults.

Developing deep multimodal fusion models to combine the MDP obtained features in order to predict actual psychological dimensions, such as emotion dysregulation, is a challenge due in part to the small nature of samples in psychology research [13].

In this work, we propose a series of methods that we hypothesize will allow us to train a scoring model for the MDP to estimate emotion dysregulation in adults. We hypothesize that such an estimation of emotion dysregulation will have psychometric convergence with the “gold standard” measure, the DERS. Our approach of choice is particularly important for the machine learning field. We hypothesize that our methodology will unleash training deep neural networks for multimodal fusion with a very small training sample.

The organization of the rest of this paper is as follows. First, we will introduce the multimodal codex, which is the heart of our approach, and the techniques required to build it and fill its missing values. Second, we will present our convolutional neural network (CNN)-transformer network architecture, including our new layer, the Feature Map Average Pooling (FMAP) layer. Third, we will discuss our training methodology. Fourth, we will present our results, including the quality of our estimation of emotion dysregulation in adults. Lastly, we will discuss these results.

## Methods

### Recruitment

#### American Subsample

This subsample consisted of 69 participants (39 females and 30 males) and was recruited online using Amazon Mechanical Turk and Prolific services between January and July 2019. The mean age for this subsample was 35.05 years (SD 12.5 years, minimum 18 years, maximum 68 years). We did not intentionally recruit any clinical participants for this subsample, but we cannot guarantee the absence of clinical patients within it.

#### French Subsample

This subsample consisted of 146 participants (88 females and 58 males) recruited between the months of January and July 2019, and was formed from multiple sources in different regions of France. Of the 146 participants, 10 clinical patients were recruited at University Hospital Center Sainte-Etienne and 22 at the Ville-Evrard Center of Psychotherapy and Psychotrauma in Saint-Denis, 33 volunteers were enrolled in Paris and 19 in Lyon, 3 college students were enrolled at Paris Descartes University and 11 at University Bourgogne Franche-Comté (Dijon), and 43 clinical private practice patients were enrolled in Paris and 5 in Lyon. The mean age for this subsample was 39.25 years (SD 13.6 years, minimum 18 years, maximum 72 years). Clinical patients were included to examine whether the MDP was capable of rightly assessing more extreme emotion dysregulation cases.

#### Tunisian Subsample

This subsample consisted of 33 Tunisian participants (21 females and 12 males) recruited in July 2019 in the city of Tunis. The mean age was 37.6 years (SD 10.5 years, minimum 17 years, maximum 55 years). While there was no intention to recruit clinical participants for this subsample, we cannot guarantee the absence of clinical patients within it.

### Measures

#### DERS-16

The original DERS [1] is a 36-item self-report questionnaire that measures an individual’s typical level of emotion dysregulation. Internally, it is based on the following 6 different subscales: (1) nonacceptance of negative emotions, (2) inability to engage in goal-oriented behaviors when in distress, (3) difficulties for controlling impulsive behaviors when in distress, (4) limited or no access to emotion regulation strategies perceived as effective, (5) lack of awareness of one’s emotions, and (6) lack of emotional clarity. Respondents have to rate items on a 5-point Likert-type scale from 1 (*almost never*) to 5 (*almost always*) depending on how much they believe each proposition applies to them. The shortened version of the DERS that we used in this work, called DERS-16 [7], consists of 16 items that assess the same 6 dimensions of emotion regulation difficulties. The total score on the DERS-16 ranges from 16 to 80, where higher scores reflect greater levels of emotion dysregulation. Importantly, this shortened version of the DERS retained excellent internal consistency, good test-retest reliability, and good convergent and discriminant validity, with only minimal differences when compared to the original DERS [7].

#### MDP

Explored in depth in an article in the Journal of Medical Internet Research [11], the MDP as a test consists of 14 themes or narratives that depict human experiences that can be either stressing or soothing in nature (loss, grief, and solitude, as well as human connection, romantic love, and kinship). The themes are evoked using rotating stimuli from a pool of pictures and short music clips that were vetted through a standardized procedure using crowd-sourced feedback. Some themes are evoked using picture stimuli alone, some are evoked using a combination of picture and music, and some are evoked by music alone (to evoke raw emotions such as sadness and fear). During the test situation, each stimulus is shown and/or heard for 15 seconds, after which the computer asks the participant to describe aloud what they have felt. They have 20 seconds to respond, before a 5-second break and then moving to the next stimulus. The whole session takes 9 minutes and 33 seconds to be completed.

Importantly, the first stimulus is fully neutral and allows us to acquire a baseline for all our measurements, which is later subtracted from them. In theory, this allows us to work with signals that react solely to the stimuli. Whether the participants came already upset to the test situation or whether they were already fatigued, the test will measure this during the first stimulus and then subtract it from the following signals; thus, it will only take into account whether a stimulus made them more upset or more fatigued, or perhaps whether a stimulus managed to soothe or relax them. The short duration of the test assures us that any abrupt changes in the signals from which the baseline was subtracted will indeed be caused by the test situation itself and not due to time simply passing by. Furthermore, the order of the stimuli themselves is such that stress and soothing themes are alternated, allowing us to get more contrast in our measurements of what each stimulus is doing to the person.

A simple way of conceptualizing the MDP is as a series of *dependent* experiments. Each stimulus intends to evoke a certain range of reactions on its own but is also linked to the reactions that the next stimulus intends to evoke. For example, stimulus 11 will attempt to provoke fear, and stimulus 12 will attempt to evoke loss, whereas stimulus 13 will evoke a soothing comforting experience of human connection. We will be interested in the reactions to each of those stimuli separately, but we will, more importantly, be interested in the relationship between them, for example, “If the person was upset by the first 2 stimuli, were they able to calm down during the last one?”

As the participant perceives the stimuli and responds aloud to them, the software automatically collects video and audio data and automatically extracts features from them. Specifically, the MDP uses an RPPG method to extract HRV features that allow measuring the sympathetic and parasympathetic branches of the autonomic nervous system; detects facial action units, head movements, and gaze direction with respect to the stimuli being presented; and analyzes speech, extracting paralinguistic features as well as conducting a linguistic analysis [13].

An important aspect of the MDP is that it does not rely on a naturalistic approach. Rather, it is based on a tightly controlled experiment carefully conceived and validated in order to evoke specific reactions.

In addition, the MDP has *content validity* [11], because it is underpinned by a strong theoretical foundation and interpretation. This sets it apart from most machine learning attempts at measuring mental health, which typically focus on prediction and convergence with a disregard for content validity [15].

Finally, contrary to most projects, wherein a machine learning system is trained to predict a category with relation to mental health, such as depressed vs not depressed, the MDP is *dimensional*. It measures psychological phenomena in terms of their continuum score, from which it is easy to produce categorical decisions (whereas the opposite is impossible to accomplish). These continuum scores are far more precise and nuanced, and could allow, among other things, to conduct outcome studies, measuring the degree of change of a psychological construct over time.

### Machine Learning Methodology

#### Important Note on Data Leakage

To prevent any form of data leaking, every step described below was conducted *within* the 8-fold cross-validation loop. This loop begins by separating the available data into a validation set and a training set containing the rest of the samples.

A few participants took the test twice at intervals of a few weeks to help with a future study on test-retest reliability, and we included both of their sessions in this study, treating them as if they were different participants. To prevent data leakage, however, when one of them was randomly put into the validation set, their other session got automatically put there as well. This explains why the validation set size changes from fold to fold (with a range of 29 to 35).

#### Data Preparation

All data preparation was performed in MATLAB 2021b (MathWorks). The MDP outputs a set of CSV files containing the structured data for each sense modality (facial expressions, linguistic analysis, etc). In most cases, this comes in the form of a table containing the timestamps as rows and the features as columns.

We averaged each feature per stimulus (ie, an average of values for facial action unit 10 from the moment stimulus 3 was shown till the moment it disappeared). We discounted the first stimulus’s results, the neutral one (see previous section), from all others so that we dealt solely with the variance produced by the test itself. Features were scaled to the −1 to 1 range, using either previous knowledge about the actual signal’s minimum and maximum values, or the empirical minimum and maximum levels found within the signal in all our training samples for a given fold.

DERS-16 scores were also linearly scaled, to the 0-1 range, to allow for quicker training times and easier interpretation of results. An important step in our data preparation procedure was to uniformize our training sample with regards to the ground truth (ie, DERS-16 scores) so that all levels of the ground truth could be equally represented in terms of the number of samples being fed to our learning algorithm. Our code did this by binning the DERS-16 score, and up-sampling our data set until all bins (ie, all score levels) had the same number of cases representing them. This, of course, presented the problem of potentially overfitting these repeated cases. In the section about test time data augmentation, we present how we dealt with this problem.

#### Multimodal Codex Sequence

From a clinician’s perspective, a typical assessment interview can be thought of as having 2 main components as follows: what is happening *at any given moment* during the interview, that is, the specific behavioral or verbal responses a patient might show to a specific question or nonverbal queue coming from the clinician, and the manner those interpreted moments *intertwine.*

Based on years of clinical experience, we argue that the psychologist or psychiatrist ends the interview with a newly acquired succession of *intuitive mental images*, representing key moments of the encounter with the patient. These mental images encode information from multiple sense modalities: a specific word that was said as well as the tone and posture in which it was said, and how that led to a long silence. They represent an utter distillation of the experience, which is the simplest representation of it.

The multimodal codex is our attempt to imitate this clinical phenomenon in a machine learning multimodal fusion context.

The multimodal codex is a grayscale computer image that encodes within it a set of meaningful multimodal features representing human responses to a controlled experiment. A multimodal codex *sequence* is the series of multimodal codexes that together encode the *flow* of the test situation.

The multimodal codex is also a practical way to encode structured tabular data in a format that can more readily be taken advantage of by CNNs. CNNs are of practical interest because (1) they ditch the need for feature engineering as they create their own features and (2) they can be trained with relatively few learnable parameters, helping to prevent overfitting.

Converting tabular data sets to images in order to use CNNs on them has been exploited by several researchers recently. Alvi et al showed that tabular data on neonatal infections could be successfully exploited using a CNN by implementing a simple transformation where features (ie, columns) are assigned, one by one, to an X-Y coordinate, with their values becoming the pixel’s intensity [16]. We will describe how we implemented their method in order to perform missing data imputation for our sample a few paragraphs below.

Buturović et al designed a tabular-data-to-graphical mapping in which each feature vector is treated as a kernel, which is then applied to an arbitrary base image [17]. Sun et al experimented using pretrained production-level CNN models implementing a diametrically opposite approach consisting of projecting the literal value of the features graphically onto an image; for example, if a feature has a value of 0.2 for a given participant in the sample, the image would include the actual number 0.2 on it [18].

The approach clearly closer to ours is that of DeepInsight [19]. Theirs is the realization that we can use a visualization technique, t-distributed stochastic neighbor embedding, in a different manner to what it was intended. While typically one applies the said technique on a data set in order to reduce the dimensions of the *feature space* to foster intuitive visualization of the sample distribution, they applied the method to their *transposed* data set, such that the *sample space* was reduced to a cartesian space for an intuitive understanding of the distribution of the *features*.

The approach we used for creating the multimodal codexes is similar, yet it differs from DeepInsight’s approach in that we implement a more modern and reliable dimensionality reduction method, the Uniform Manifold Approximation and Projection (UMAP) [20]. Its strength is to better preserve the global structure of the data and thus the relationship between the features. In addition, we apply this procedure to a very specific kind of tabular data (multimodal sensing data). To the best of our knowledge, this has not been proposed before.

Our proposed method to missing data imputation can be described by the following pseudocode: *For each feature in the data set, (1) produce an image by disposing each feature vector in the dataset, EXCEPT the current one, as pixels in a grayscale image, with the intensity of the feature representing the pixel’s intensity; (2) feed the created picture for each participant to a simple CNN consisting of 2 convolutional layers and a dense layer, the mission of which is to find visual patterns in the projected data that can predict the left-out feature; and (3) use the created model to predict the missing values corresponding to that feature.*

For each fold, we learn the missing data imputation models from the learning set and fill with it the missing values of both training and validation sets.

Our proposed process to create a multimodal codex sequence is resumed in the following pseudocode: *For each of the 13 stimuli, (1) group all features corresponding to a given stimulus in the form of a SAMPLES × FEATURES matrix; (2) use the UMAP method over the transposed matrix to obtain the X and Y coordinates for each feature; and (3) create a 28×28 pixel grayscale image per person, printing the value of each feature in their respective X and Y coordinates.*

**Figure 1:**
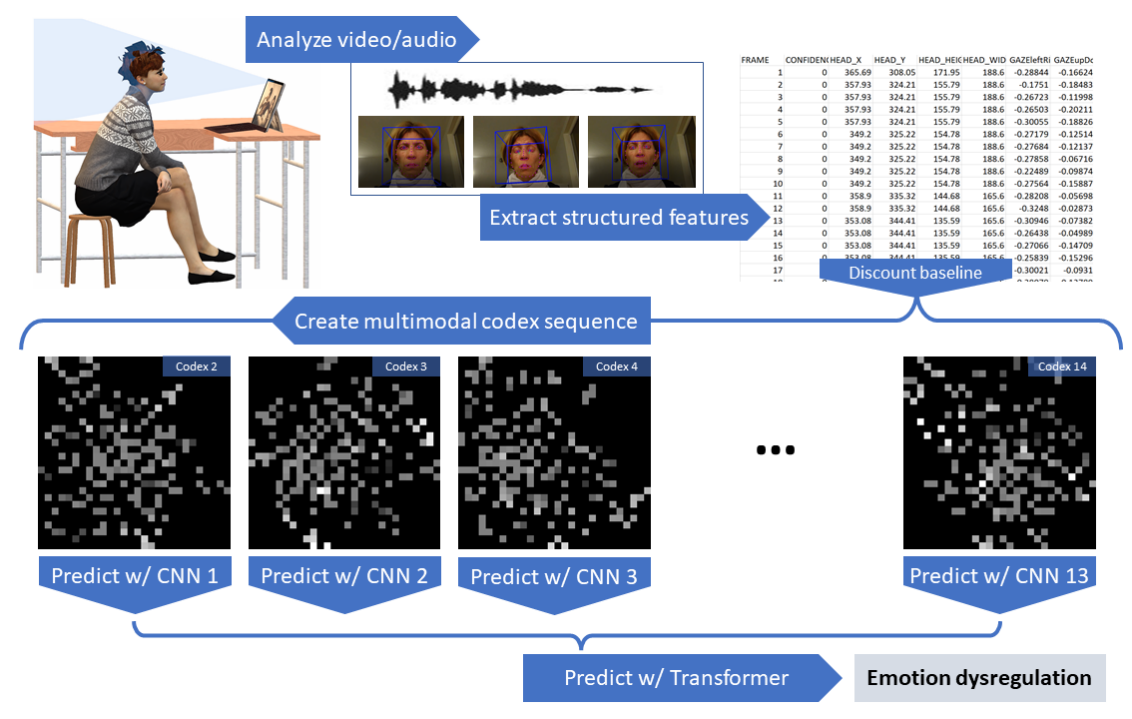
From test to result. Top left: a woman taking the Multimodal Developmental Profile test. Top center: the audio wave and video frames, with the latter showing the analysis for head pose, eye gaze, and facial expressions. Top right: tabular data of some of the features extracted from the audio and video. Bottom: the 2nd, 3rd, 4th, and 14th multimodal codexes for a participant in the sample. CNN: convolutional neural network; w/: with.

The resultant images look like those in Figure 1.

This process naturally builds images with distinct clusters of features for each stimulus depending on the specific relationship between the typical responses to the said stimulus in the sample and the ground truth variable. Like a clinician’s intuition described earlier, our approach could end clustering together a series of language markers, facial expressions, and HRV features, which might not initially be obvious, in the context of what is evoked by a specific stimulus and the typical response pattern in the sample.

Practically, this takes the guessing out of feature engineering, while also providing the CNNs with smaller clusters to “look at,” which in turn puts less stringent requirements on the *receptive field* of the network, leading potentially to smaller kernels and fewer layers.

An important limitation of UMAP and all other visualization techniques of the sort is that the proximity of points in the projection they generate does not follow a predictable pattern. While points that are closer together typically are more related than those projected far away, this is not guaranteed for all cases, and the relationship between distance and importance is certainly not linear.

On occasion, the mapping for two or more features falls in the exact same X and Y coordinates. While this could be easily remediated by enlarging the codex resolution, we decided to leave this as a feature. When UMAP considers 2 features to be so close, they might as well mean the exact same thing. In that case, we average the value of the features to find the value of the pixel in question.

For each fold, we learned the mapping from the learning set and created with it the multimodal codexes for the learning and validation sets.

#### Multimodal Fusion Network Architecture

As described in the previous section, the problem of assessing a psychological construct during an interview is both a spatial problem (ie, measuring different things that happen simultaneously) and a temporal problem (understanding the succession of events and their relationship).

For dealing with the first part of the problem, we implemented 13 CNNs, with 1 per stimulus (minus the baseline stimulus). The reason not to rely on just 1 network for all of the stimuli is that we do not assume the features that are important to predict emotion dysregulation are the same during each stimulus response. On the contrary, a clinician will look for specific patterns in the patient’s behaviors depending on the queue the therapist has sent right before during the interview. Patterns can actually reverse. A cluster of features indicative of emotion dysregulation given 1 stimulus can actually be indicative of good regulation during another.

We confronted the following challenges when designing the architecture for our CNNs: (1) How to create a deep enough network that will be able to extract complex concepts, while keeping the number of learnables (ie, weights) very lean to avoid overfitting (ie, memorizing) our small training set? (2) How to avoid downsampling/blurriness of the codex when going deeper into the network, a classic byproduct of pooling layers, so that deeper layers can still take advantage of details while simultaneously uncovering more global patterns? To overcome these challenges, we implemented cutting-edge best practices as well as some innovations.

**Figure 2:**
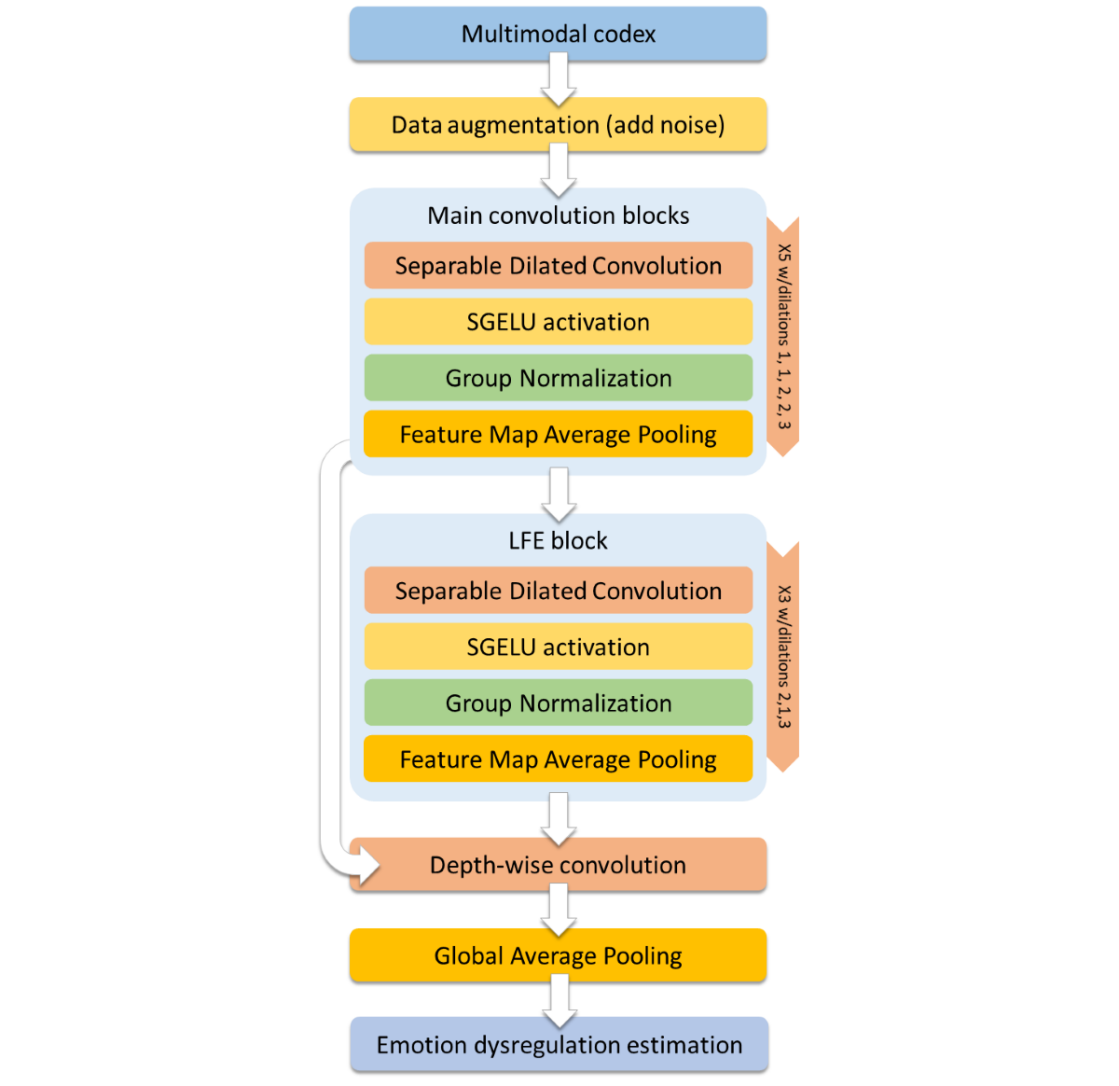
Our convolutional architecture (339 weights). LFE: local feature extraction; SGELU: Symmetrical Gaussian Error Linear Units.

The network begins with a multimodal codex augmentation layer that we will explore later. The rest of the network is basically constituted of 8 convolutional blocks, each containing a depth-wise separable convolution layer [21] with 8 3×3-sized kernels, with different dilation factors (more below), a stringent L1-L2 norm weight-decay regime, and a constrained range of values for the weights to take, lying between −1 and 1; a mean-shifted Symmetrical Gaussian Error Linear Units (SGELU) [22] activation layer; a group normalization layer [23]; and our new FMAP layer (details are presented in the next section). There is a residual connection that allows gradients to flow directly from the end of the network toward the output of the 5th convolutional block. After adding the residual and the upcoming connection from the last convolution block, the network ends with a depth-wise convolution layer (ie, kernel 1×1), a linear activation layer, and a Global Average Pooling (GAP) [24] layer. The whole CNN can be seen in Figure 2 (all 13 networks share identical architecture). It has only 339 weights overall.

Importantly, our proposed architecture dispenses with pooling layers entirely. They are typically used as a means to increase the effective receptive field when moving deeper into the network. They were replaced with a carefully calculated set of kernel dilation factors, which increase from the 1st block to the 5th, then decrease for blocks 6 and 7, and then increase once again in block 8 before the network ends. This decrease and increase between blocks 6 and 8 is what Hamaguchi et al have called a local feature extraction (LFE) module [25]. In their important work on satellite imagery, they have shown that in scenarios where both general patterns and details are important for prediction, reducing and then rapidly increasing the dilation factor can allow the network to take into account both detail *and* structure all the way to the deepest layers of the network. In our case, this is crucial, because although we trust the thinking behind the multimodal codex design, the UMAP method is not infallible, and a very important feature to predict emotion dysregulation might still end lying away (graphically) from the main clusters, as a single pixel somewhere in the image, that would tend to disappear when down-sampled. Different from the approach by Hamaguchi et al, though, we included a residual connection going from block 5 (right before entering the LFE module) directly into the last block, basically short-circuiting the LFE module. This allows our network to decide during training if the module is needed or not, depending on the actual data correlations it finds, and even to find the right balance of detail and structure automatically. The dilation factor of each convolutional layer was carefully calculated so that the *effective* receptive field covers the whole image (28×28) by the end of the network.

In the following paragraphs, we provide a brief description of each of the components of the network as well as the rationale behind their implementation in the context of deep learning from small data sets.

Depth-wise separable convolutional layers were first introduced in a previous study by Chollet et al [21] and implemented in Google’s Xception and MobileNet architectures. A depth-wise separable convolution separates the convolution process into the following 2 parts: a depth-wise convolution, and a pointwise convolution. They can allow for a reduction of parameters of up to 95% compared to classic convolutional layers [26]. While this reduction is typically desired from the perspective of lessening computational and size demands of neural networks, particularly during prediction time and for mobile hardware deployment, our rationale for using them is entirely different. In classical statistics, it is known that small samples should be fitted with models using relatively few degrees of freedom (ie, parameters) if one wants to prevent overfitting the training set. Typically, the best practice ratio is 10 to 1; ie, 10 times fewer degrees of freedom than data available. While that ideal might be too stringent when ported to modern machine learning, we still thought it was vital to keep it as a guiding principle. The fewer parameters we used, the least the network *could* overfit the data. Hence, our utilization of these layers.

SGELU activation was recently introduced in a previous study by Yu et al [22]. Yu et al took advantage of the already powerful GELU function, which represents nonlinearity by using the stochastic regularizer on an input (the cumulative distribution function derived from the Gaussian error function), which has shown several advantages over other activation functions and is currently implemented in modern natural language processing (NLP) transformer models. The new SGELU function allows activations to take on equally large negative and positive values, pushing the weights to also do so. In their investigation, they found that this new activation function performs better than all other available activation functions, but this was not the reason that had us choose it for our task. Rather, they also reported that training becomes smoother and more stable when using SGELU and that they found preliminary evidence of better generalization of the network when trained with it. Since ours is a task that deals with a very small data set and thus probably exaggerated levels of variance, smoother more stable training can be crucial, and the capacity to generalize better could indicate greater self-regularization, which is essential when learning from a small sample.

Mean shifting [27] is a method that consists of simulating random data, similar to what an activation function might compute, and passing it through the activation function, in our case SGELU, to find the empirical mean of the activations. Once we find it, we can subtract it from 0, the desired mean for the activations, and then add (ie, shift) that difference to the activation itself. In so doing, now the empirical mean of the activation function becomes 0 (for random data). This approach has been shown to increase both convergence speed and accuracy.

Group normalization was introduced by the Facebook AI Research (FAIR) team in 2019 [23]. Its claim to fame was its capacity to produce performance results that paralleled batch normalization when using regularly sized batches, but that strongly outperformed it when using small batches. Small batches are more typical in the context of parallelization of neural networks training within computing clusters. Although we also got interested in it because of its capacity to deal with small batches, our reasoning was not computational. Instead, it has been shown that smaller batches increase regularization by, among other things, increasing stochasticity [28,29]. Importantly, we implemented group normalization *after* the SGELU activation functions for the following reason: as reported by [22], if activations are normalized *before* they hit the SGELU activation function, there is a risk that the full extent of it might not be used, particularly the nonlinear nature of both extremes of the function. We hard-coded the group norm hyperparameter, which decides the number of groups, to be always half of the number of kernels in the previous CNN layer (so 4 for all of our blocks).

The networks end with a GAP [24] layer to average the final activation map; the result of that operation is the prediction of the network. The GAP layer has come to replace fully connected layers in CNNs lately, mainly because of its capacity to reduce overfitting and drastically reduce parameters.

The full CNN model is shown in Figure 2.

After each of the 13 CNNs produce an estimation of emotion dysregulation, those estimations become the sequential data fed to the next and final architecture, to deal with the temporal aspect of our problem, which is the transformer.

Endowed with the task of decoding the sequential meaning of the participant’s responses to the succession of MDP’s controlled experiments, our transformer network is of course inspired by the seminal work of Vaswani and the team at Google Brain [30]. Transformers have replaced recurrent neural networks and their convolutional counterparts for an ever-increasing number of sequential learning tasks, including NLP, video classification, etc. Indeed, they can be trained faster than models based on recurrent or convolutional layers [30].

At their core is the multiheaded attention mechanism, which allows evaluating, in parallel and for each data point in a sequence, which other data points in the said sequence are relevant to the assessment. The attention heads in our encoder block are of size 13, to cover the whole MDP sequence, as opposed to the size of 64 used in the study by Vaswani et al, and we used 4 heads as opposed to 8. Our encoder block also includes residual connections, layer normalization, and dropout. The projection layers are implemented using a 1D convolution layer.

The encoder was followed by a 1D GAP layer to reduce the output tensor of the encoder to a vector of features for each data point in the current batch. Right after this is the multilayer perceptron regression head, consisting of a stack of fully connected layers with ReLU activation, followed by a final 1 neuron–sized fully connected layer with linear activation that produces the actual estimation of emotion dysregulation. We tried implementing positional encodings, as per the original paper, as well as look-ahead masking; however, both methods yielded worse results for our use case, so we discarded them.

In the original paper, Vaswani et al implemented label smoothing. Given that ours is a regression problem, we switched this for test-time augmentation (TTA), which will be described later.

**Figure 3:**
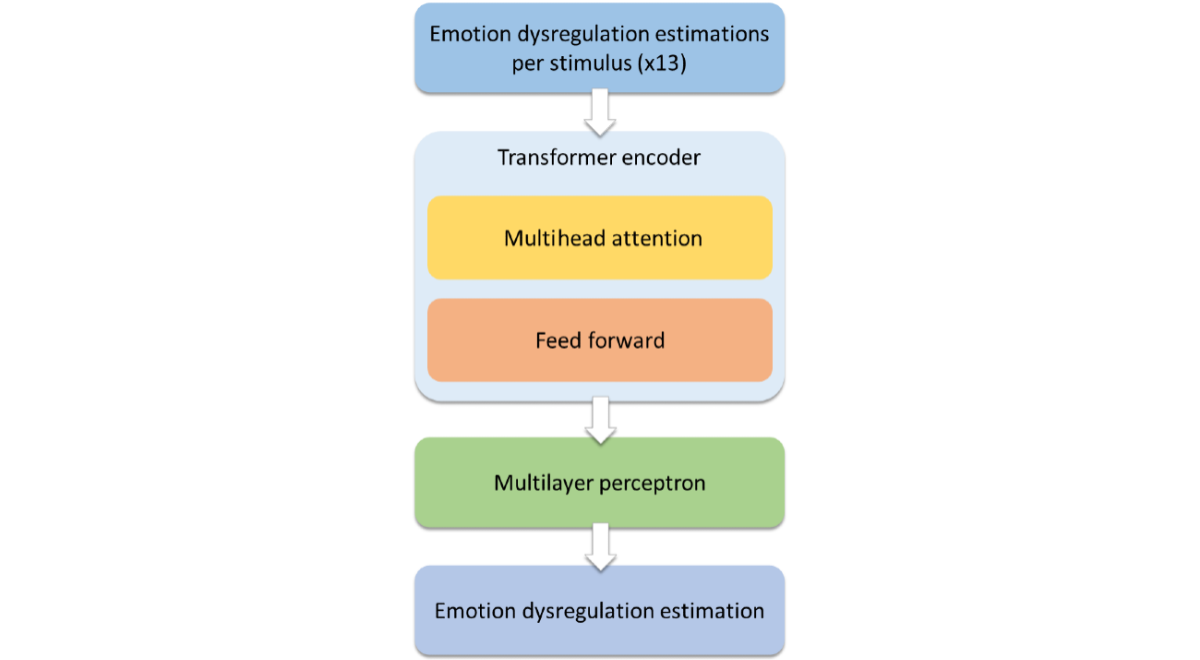
Our transformer architecture (4223 weights).

The loss function for our transformer architecture was the concordance correlation coefficient (CCC) [31]. It was pioneered as a loss function by Atmaja et al, and tends to find a good balance of low error and high correlation between predictions and the ground truth [32]. Our transformer architecture can be seen in Figure 3.

#### FMAP Layer

This new kind of layer computes the average of the activations or feature maps produced by a 2D convolution layer as follows:

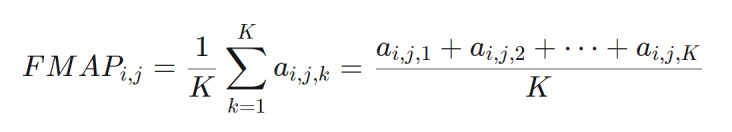



where *a* is a 3D “channels-last” tensor and *K* is the number of kernels of the previous convolution layer (ie, the number of channels).

It was inspired by the GAP layer, which revolutionized CNNs by drastically reducing the number of weights without sacrificing performance, while increasing regularization. However, the FMAP layer averages tensors among feature maps (ie, channels), as opposed to across the 2 dimensions of each feature map like GAP does.

If included at the end of every convolutional block, FMAP assures that the depth (ie, number of channels) of the activations flowing forward in the network remains flat (ie, 1 channel) at all depths of the network, instead of exponentially increasing, as is typically the case.

It is important to realize that a sort of weighted average *already* happens within regular convolutional layers when they calculate the dot product (ie, cross-correlations) between the kernel weights and the image pixels for each of its channels. By analogy, with FMAP, we are transforming that into a nonweighted average.

The FMAP can also be thought of as a nonlearnable version of the depth-wise convolution (ie, convolutions with kernel size 1×1 typically used to reduce the complexity of a model by merging its feature maps). By using a fixed function (average) instead of a learned one, though, we obtain a decrease in learnable weights in our model. For a depth-wise convolution, we need 1 weight and 1 bias per input feature map, whereas with FMAP, we need none. We also prevent the network from overfitting the training set during the computation.

In terms of the decrease in the number of weights for a network, in our own CNNs, the reduction is of 71% (from 1172 weights to 339). This remarkable reduction in weights has several effects, including reducing computational demands for both training and prediction, and, as we mentioned earlier, reducing the number of degrees of freedom in the model, thus reducing the potential to overfit the training set.

We believe this layer forces an ensembling effect onto the network’s block in which it is inserted. It is a consensual observation that ensembles of trained neural networks generalize better than just 1 trained neural network [33]. This is because their different random initializations increase stochasticity, empowering each network in the ensemble to explore the loss landscape by taking entirely different paths toward minima, and when their predictions are averaged, they can cancel each other’s overfitting tendencies out. We think that when FMAP layers are used consistently after all (or at least many) 2D convolutional layers, the same ensembling effect is introduced *within* subnetworks (ie, blocks) of the network, so that each block ending in an FMAP layer is forced to create an ensemble of subnetworks. This, we hypothesize, should introduce desirable block-wise stochasticity that increases model generalization ability without the need to train multiple entire neural networks.

#### Training and Test Time Data Augmentation Scheme

In our quest against overfitting, we implemented data augmentation. In its classic form, it allows for the on-the-fly creation of new training examples based on random transformations of the original ones.

With regard to our CNNs, we created a layer designed to introduce uniform random noise within the multimodal codexes. During training, it introduces up to 10% noise for each pixel representing a feature in the multimodal codex (while it leaves all other pixels, the ones not representing any feature, alone). This meant that, for each epoch, the network saw an up to 10% different version of each image.

This procedure was especially important given that our uniformization of the ground truth variable by upsampling meant that there was a nonnegligible amount of image (multimodal codex) repetition being fed to the CNNs. So this data augmentation scheme allowed for them to be actually *somewhat* different.

Another more modern form of data augmentation is TTA [34]. This approach consists of, at prediction time, generating on the fly X-augmented data sets, predicting with each, and then averaging the results.

The way we implement TTA is innovative. We use it between our spatial (CNNs) and temporal (transformer) networks. When our 13 CNNs predict their final emotion dysregulation estimates, we do so using TTA, and moreover, we repeat the process 10 times. As a result, we provide the transformer with both better predictions and more diverse data to train on. We believe this procedure can greatly increase the generalization of the network to unseen data.

#### Training Procedure

We used vanilla Adam optimizer for both our CNNs and the transformer network, with default settings. We did not implement any learning rate scheduler.

We trained our CNNs for 500 epochs each. We trained our transformer network for 100 epochs. At each epoch, the models were saved. By the end of training, our code automatically selected the best model, which was the one with the highest Pearson correlation for our CNNs and that with the highest CCC for our transformer, between predictions and the ground truth on the validation set.

As we described earlier, all the aforementioned steps were implemented within each fold of a cross-validation procedure. Eight folds were utilized overall.

### Analyses

Pearson correlation coefficient was calculated using SciPy, version 1.7.1 (Community Library Project). Mean absolute error and the CCC were assessed using Tensorflow, version 2.6.0 (Google Brain; code included in the associated Google Colab, see section below). Means and standard deviations were calculated using NumPy, version 1.19.5 (Community Project).

#### Convergent Validity Analysis and Interpretation Criteria

Convergent validity is the extent to which a measure produces results that are similar to other validated measures measuring the same construct [35]. A standard way of measuring it is by using Pearson product moment correlation [36]. We will interpret Pearson’s results based on a review by Drummond et al on the best practices for interpreting validity coefficients, where a value ≥0.5 indicates very high correlation, 0.4 to 0.49 indicates high correlation, 0.21 to 0.4 indicates moderate correlation, and ≤0.2 indicates unacceptable correlation [37].

### Replicability via Google Colab

We decided to port a large portion of our work from MATLAB to Tensorflow/Keras (created by François Chollet) and to prepare a Jupyter Notebook within Google Colab so that every reader can replicate our findings. The notebook can be accessed online [38]. It can be executed on Colab itself, or downloaded and run locally.

## Results

**Figure 4:**
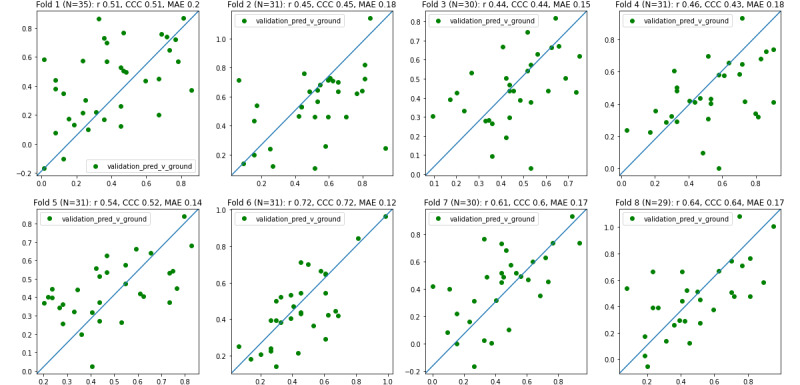
Scatter plot. Prediction (ie, estimation) vs Difficulties in Emotion Regulation Scale, brief version (DERS-16) for each fold. Pearson *r*, concordance correlation coefficient (CCC), and mean absolute error (MAE) are provided for each fold.

**Figure 5:**
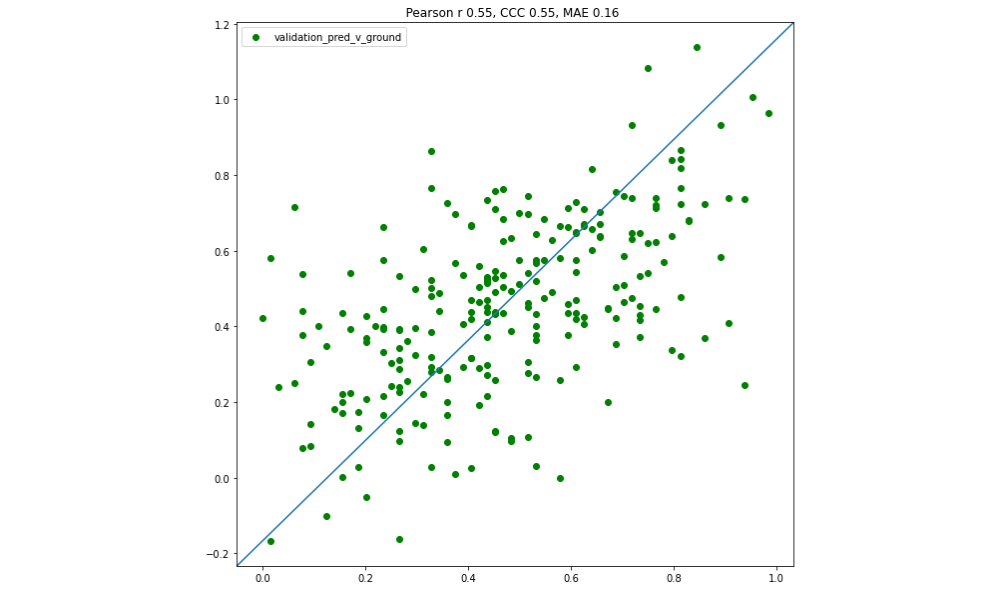
Eight folds’ validation sets combined (N=248). Pearson *r*, concordance correlation coefficient (CCC), and mean absolute error (MAE) are provided for this combined sample.

The results are presented in Figure 4, Figure 5, and Table 1.

**Table 1:** Data per fold for our system’s estimated emotion dysregulation versus the findings with the Difficulties in Emotion Regulation Scale, brief version (DERS-16; ground truth).

Variable		Number	Pearson *r*	*P* value	CCC^a^	MAE^b^
**Fold**						
	1	35	0.51	.002	0.51	0.20
	2	31	0.45	.01	0.45	0.18
	3	30	0.44	.01	0.44	0.15
	4	31	0.46	.01	0.43	0.18
	5	31	0.54	.002	0.52	0.14
	6	31	0.72	<.001	0.72	0.12
	7	30	0.61	<.001	0.60	0.17
	8	29	0.64	<.001	0.64	0.17
Mean value^c^		N/A^d^	0.55	<.001	0.54	0.16
SD value^e^		N/A	0.10	.01	0.10	0.02

^a^CCC: concordance correlation coefficient.

^b^MAE: mean absolute error.

^c^The mean across folds for each metric.

^d^N/A: not applicable.

^e^The mean of the standard deviations across folds for each metric.

## Discussion

### Principal Findings

Can computers detect emotion dysregulation in adults, by looking at their behavior and physiology during a set of controlled experiments? Can they generate “mental images” containing different sense modalities, like clinicians do? Can they do so in a sample that spans different cultures and languages? Can one train a deep multimodal fusion neural network using only a couple of thousand parameters? These are some of the questions we set out to answer in this work. This study evaluated the convergence validity of MDP’s emotion dysregulation estimation with regard to DERS-16, a brief version of the “gold standard” measure for emotion dysregulation. We interpret our results as excellent evidence for convergence validity between MDP’s emotion dysregulation estimation and the DERS-16 in our sample, suggesting that scores obtained using the MDP are valid measures of emotion dysregulation in adults.

It is important to reflect on the diversity of our sample. It spanned 3 continents and 2 languages, with a broad age range, and included individuals with psychopathology to represent the higher end of the emotion dysregulation spectrum. With that in mind, we believe it is impressive that emotion dysregulation estimations were so correlated with their DERS-16 counterparts for all folds, showing similar results. We think this shows a preliminary form of cross-cultural validity for the approach, adding to the evidence we found in our prior work [13]. It also shows that the MDP is capable of assessing emotion dysregulation in adults with a psychopathology.

We think the multimodal codex approach captures quite well the mental processes that occur in the mind of a clinician while conducting an assessment interview. We attribute the success of our approach in large part to the good framing of the problem as spatiotemporal, and believe this representation of all sense modalities as a combined image is closer to the way we humans do multimodal fusion.

To our knowledge, the MDP is the first test of its kind. It is a validated exposure-based psychometric test that implements deep multimodal fusion to analyze responses within a set of controlled experiments in order to measure psychological constructs.

Its advantages over classical questionnaires and interview-based tests are manifold. They are as follows: the MDP takes less than 10 minutes to complete; it can be taken at home with a computer or tablet and is resilient to unpredictable variability in the test conditions; it is scored automatically in minutes; it is objective and replicable in its observations; it is holistic, taking into account language, voluntary and involuntary behavior, and physiology; it can be used in different cultures with only minimal translation efforts; and it can evolve over time, learning new scoring models based on different validated psychometric measures.

In terms of deep learning, we cannot stress enough how this work defies current trends and tenets within the field. In the current international race toward the trillion-parameter model, how can anyone dare to present a deep network capable of estimating very abstract psychological phenomena with only 8630 weights? In a field powered by Google, Apple, Facebook, Amazon, and other American and Asian tech giants data mining free online services for millions of data points, how can anyone dare to present a model that can be well trained with only 274 examples? We think this work should be seen as pertaining to a concurrent and perhaps literally opposite trend. Humans do not need that many examples to learn something, even something complex. Maybe machines do not need it either, provided intelligent constraints are put in place (sort of bike wheels for children) to prevent the system from falling into tendencies (memorization, ie, overfitting) that would prevent real learning. We think that at the heart of this concurrent view of machine learning, there is chaos in the form of randomness. Random noise has been added to our samples as data augmentation. There are random paths toward minima spearheaded by an increase in stochasticity due to small batches during training. There is randomness during prediction by implementing TTA. There is randomness in the random initialization of each kernel within each convolutional block, and the way the FMAP layers force them to ensemble. There is randomness in the automatic choice of the stimulus from the stimuli pool so that no single person experiences the exact same stimuli set. There is randomness in the random errors that occur in pretty much every one of the feature extraction processes implemented by the MDP software. Randomness might seem to be just noise, but what if, in reality, it is what allows us to separate signal from noise?

### Limitations and Future Directions

One of the obvious limitations of our work is the size of our sample. Although we purposely set to prove that one can learn very complex and deep multimodal models that can be accurate and reliable with just a few hundred cases, this does not in any way disprove the common sense assumption that, with more data, the model would improve even more. In addition to sheer sample size, we believe it would be interesting, and quite unexplored in psychometry, to use census-based samples (data sets whose distribution in terms of sex, age, income, etc, matches the census of a given country). Online recruiting agencies are beginning to propose this as a service, and we hope we will be able to work with such a sample in the near future.

Another weak point of our study is the lack of a hold-out test set. We did not implement one primarily because of a lack of enough data. Indeed, it is known that validation sets can be overfitted, in a process some have called “model hacking” [39]. Model hacking is the extensive repetition of a cross-validation scheme for hyperparameter tuning and model development, for which we report only the best fit found. Similar to “human overfitting,” our resulting model might obtain great cross-validation scores but perform more poorly in new unseen samples. This is especially true with brute-force approaches to hyperparameter tuning. Small-sized samples, such as ours, that contain high variability and an extremely diverse population are somewhat inherently protected against model hacking. Each fold’s validation set will be strongly different from that of another fold, not to mention that training samples themselves will be very different from fold to fold, producing quite different models. If with such variability the model still shows stable performance across all or most folds, it might be a good indication that the methodology and the models resulting from it do generalize well. In addition, we took some empirical measures to prevent model hacking, such as having a random seed set at the beginning of our code, so that the partition of folds was always equal, and then working with the first fold for hyperparameter tuning and model tuning. Most importantly, we have not implemented any sort of automatic search algorithm for hyperparameter tuning. Instead, we chose to explore only a handful of theoretically promising options by hand.

Furthermore, we question whether a hold-out sample, proportional in size to our overall sample, would have been a better unbiased estimator (how can a sample with a size of around 30 be taken as representative of the whole population?). In the future, we will look to the works of Martin and Corneanu [40,41] that unlock estimating generalization performance directly from the characteristics of the model itself. We are already working on a criterion inspired by their work, which we call the network engagement criterion. This criterion seems promising in estimating test error using only the training sample. Such a method would, in our opinion, close the circle, completing the set of methods and approaches we presented in this work to fully implement a cycle of unbiased learning with the sort of “small data” samples commonly found in the social sciences.

### Conclusion

In this work, we successfully trained a deep neural network consisting of spatial (convolutional) and sequential (transformer) submodels, to estimate emotion dysregulation in adults. Remarkably, we were able to do so with only a small sample of 248 participants, without using transfer learning. The metrics of performance we used show not only that the network seems to generalize well, but also that its correlation with the “gold standard” DERS-16 questionnaire is such that our system is a promising alternative. Perhaps most importantly, it was confirmed that deep learning does not need to mean millions of parameters or even millions of training examples. Carefully designed experiments, diverse small data, and careful design choices that increase self-regularization might be sufficient.

## Abbreviations

BAT: Biometric Attachment Test
CCC: concordance correlation coefficient
CNN: convolutional neural network
DERS: Difficulties in Emotional Regulation Scale
FMAP: Feature Map Average Pooling
GAP: Global Average Pooling
HRV: heart rate variability
LFE: local feature extraction
MDP: Multimodal Developmental Profile
NLP: natural language processing
RPPG: remote photoplethysmography
SGELU: Symmetrical Gaussian Error Linear Units
TTA: test-time augmentation
UMAP: Uniform Manifold Approximation and Projection
